# Effects of indigestible carbohydrates in barley on glucose metabolism, appetite and voluntary food intake over 16 h in healthy adults

**DOI:** 10.1186/1475-2891-12-46

**Published:** 2013-04-11

**Authors:** Elin V Johansson, Anne C Nilsson, Elin M Östman, Inger M E Björck

**Affiliations:** 1 Division of Applied Nutrition and Food Chemistry, Department of Food Technology, Engineering and Nutrition, Lund University, P. O. Box 124, Lund, SE-221 00, Sweden

**Keywords:** Barley, Dietary fibre, Glucose tolerance, Incretins, GLP-1, Energy intake, Appetite, Colonic fermentation, Metabolic syndrome, Inflammation

## Abstract

**Background:**

Recent knowledge in animals suggests that gut microbial metabolism may affect host metabolism, including appetite regulating hormones. The aim of the present study was to evaluate the potential effects of a whole grain barley kernel product, rich in intrinsic indigestible carbohydrates (dietary fibre and resistant starch), on markers of metabolism and appetite regulation in healthy subjects.

**Methods:**

Boiled barley kernels (BK) or white wheat bread (WWB; reference) were provided as late evening meals to 19 young adults in random order using a cross-over design. During subsequent ad libitum standardized breakfast and lunch meals (10.5-16 h), blood was collected for analysis of glucose, plasma insulin, adiponectin, ghrelin, glucose-dependent insulinotropic polypeptide (GIP) and glucagon-like peptide-1 (GLP-1), serum free fatty acids (FFA) and interleukin (IL)-6. In addition, appetite sensations, voluntary energy intake and breath H_2_ were determined.

**Results:**

BK as evening meal increased plasma GLP-1 at fasting (*P* < 0.05) and during the experimental day (*P* < 0.01) compared with WWB. In addition the BK evening meal decreased fasting serum FFA (*P* < 0.05) and tended to decrease fasting serum IL-6 (*P* = 0.06). At lunch, preceded by BK evening meal, voluntary energy intake was decreased (*P* < 0.05) when compared to WWB evening meal. The BK evening meal decreased incremental blood glucose area (*P* < 0.01), promoted higher breath H_2_ (*P* < 0.001), maintained adiponectin concentrations (*P* < 0.05) and reduced perceived hunger (*P <* 0.05) during 10.5-16 h after the meal.

**Conclusions:**

The results indicate that the BK evening meal, facilitate glucose regulation, increase the release of GLP-1, reduce subsequent energy intake while at the same time decreasing hunger over 2 subsequent meals, and reduce fasting FFA the subsequent morning, possibly mediated through gut microbial fermentation of the indigestible carbohydrates.

## Background

The metabolic syndrome (MetS) represents a cluster of risk factors identifying subjects at high risk of developing type 2 diabetes (T2D) and cardio-vascular disease [1]. The prevalence of obesity and T2D is increasing globally and the World Health Organization estimates in 2012 the number of people suffering from T2D worldwide to be 347 million, and that the number of diabetes deaths will increase by two thirds between year 2008 and 2030 [2]. The need for preventive strategies is thus urgent.

Observational studies have shown that higher intake of whole grain (WG) is associated with lower body mass index (BMI) [3-5], improved insulin sensitivity [6], reduced risk of T2D [7] and of premature total and cause-specific death [8]. Several mechanisms have been discussed as mediators of favorable metabolic outcomes following WG intake, e.g. low GI features, presence of dietary fibre (DF) per se and/or the presence of DF associated bioactive components [9-12]. It has been hypothesized that dietary factors may affect composition and metabolism of the gut microflora [13,14], and a close connection between the microbial composition and inflammatory status has been observed [15,16]. Consequently, metabolic derangement and also obesity has been described as an endotoxemic inflammatory condition triggered by e.g. high fat feeding [17,18]. Oligofructose has been shown to increase bifidobacteria in obese mice [19], which was accompanied by improved glucose regulation and a reduced inflammatory tonus [20,21], indicating a prebiotic mechanism. Less is known about possible colonic mediated effects of DF present in WG diets. A relation between WG intake and improved inflammatory status was reported in a cross-sectional study in healthy subjects [22]. In addition, certain WG products, rich in cereal DF and resistant starch (RS) facilitated blood glucose regulation and improved inflammatory tonus in humans in the perspective from a late evening meal to a standardized breakfast, i.e. in the time frame 10 to 12 h after meal [23]. The metabolic benefits has been proposed to be associated with colonic fermentation of indigestible carbohydrates (DF and RS) [24-26], and an associated increase in systemic levels of glucagon-like peptide-1 (GLP-1) [23]. GLP-1 is increasingly being assigned both anti-diabetic and anti-obesogenic features [27-29], which makes it relevant to perform further studies regarding the possible relation between food derived stimulation of this incretin and effects on metabolic parameters.

The objective of this work was to evaluate the effect of intrinsic indigestible carbohydrates in boiled barley kernels (BK) consumed in the evening on glycaemia, appetite sensations, appetite regulatory hormones and voluntary food intake, at fasting and in the post-prandial phase following two consecutive meals (breakfast and lunch). Variables determined in blood were glucose, insulin, GLP-1, glucose-dependent insulinotropic polypeptide (GIP), ghrelin, free fatty acids (FFA), adiponectin and interleukin (IL)-6. In addition breath hydrogen (H_2_) excretion was determined as a marker of colonic fermentation. The breakfast and lunch meals were provided ad libitum, allowing for evaluation of over-night effects on metabolism and appetite regulation in a realistic eating situation. For this purpose, carbohydrate-equivalent meals, consisting of boiled BK or white wheat bread (WWB, reference meal), were provided as evening meals to healthy subjects, using a randomized cross-over design.

## Materials and methods

### Subjects

Nineteen healthy volunteers, 6 men and 13 women aged 24.2 ± 1.9 years, with normal body mass indices (BMI) (mean ± SD = 22.3 ± 2.0 kg/m^2^) participated in the study. The inclusion criteria were age between 20 – 35 years, BMI between 18 – 25 kg/m^2^, non-smoker and no known metabolic disorders or food allergies. Approval of the study was given by the Regional Ethical Review Board in Lund, Sweden (Reference 668/2008).

### Evening test- and reference meals

The test- and reference meals were based on 50 g potentially available starch.

*BK, test meal*; One portion (96.8 g) of slightly polished, dried barley kernels (Finax, Helsingborg, Sweden) was boiled for 20 min in 150 ml water containing 0.25 g NaCl. All water was absorbed into the kernels. The BK had the appearance of a rice-analogue and was consumed with 250–300 ml water.

*WWB, reference meal;* The WWB was baked according to a standardized procedure in a home baking machine (Severin model nr. BM 3983; Menu choice, program 2 [white bread, 1000 g, quick (time2:35)]). The bread was made from 540 g of white wheat flour (Kungsörnen AB, Järna, Sweden), 360 g water, 4.8 g dry yeast, 4.8 g NaCl. After cooling, the crust was removed and the bread was sliced and portions (119.7 g bread) were wrapped in aluminum foil, put into plastic bags and stored in a freezer (-20°C). At the day of consumption the subjects were instructed to thaw the bread at ambient temperature, still wrapped in aluminium foil and in the plastic bag. The WWB was consumed with 250–300 ml water.

### Ad libitum breakfast and ad libitum lunch

The breakfast consisted of commercial, low fibre, high glycemic index white wheat bread (Dollar Storfranska, Lockarp, Malmö, Sweden), butter (BreGott, Arla Foods, Stockholm, Sweden) and ham. The sandwiches were cut in small pieces (6.5 × 6.5 cm), served as double-sandwiches whole or cut diagonally. The subjects were supposed to choose freely the amount ingested. The breakfast was served with 300 ml water.

The lunch consisted of Swedish hash i.e. fried mix of diced potato, meat and onions (Felix Krögarpytt, Procordia Food AB, Eslöv, Sweden). If ketchup (Felix, Procordia Food AB, Eslöv, Sweden) was chosen to be consumed with the hash, the subjects was obliged to maintain the amount of ketchup at both lunch situations. Water was served with the lunch (250 ml).

### Study design

The design was randomized cross-over. BK and WWB were included as late evening meals (0930 pm), separated by approximately 1 week. Each evening meal was consumed twice, meaning that the test subject participated at four separated occasions. Fasting measurements were performed at all four visits and postprandial measurements were performed at two of the visits (randomly chosen), one visit after BK and WWB, respectively. At the days for postprandial measurements, test subjects were provided ad libitum intake of breakfast and lunch, and physiological test variables were repeatedly measured during the experimental day. Blood glucose, breath H_2_, visual analogue scale (VAS) ratings for subjective appetite (hunger, satiety and desire to eat) and samples for measurements of insulin, active ghrelin, total GIP, and active GLP-1 were obtained at fasting and 15, 30, 45, 60, 90, 120, 180, 210, 225, 240, 255, 270, 300 and 330 minutes after commencing the breakfast. Samples for IL-6 and adiponectin were collected at 0, 60, 120, 210, 270 and 330 minutes and measurements of FFA were performed at time 0 and 210 min.

### Procedure

The subjects were encouraged to standardize their meal pattern and to maintain their regular eating habits during the experimental period. They were also instructed to avoid alcohol, excessive physical exercise or food rich in DF the day prior to the evening test or reference meals. Furthermore, they should not have taken antibiotics or probiotics during the previous 2 week period. At 0930 pm the evening before each experimental day, the subjects prepared and consumed evening meals in their home. The WWB was distributed as frozen portions; and the uncooked BK were provided in portions ready to cook. The BK meal was prepared according to a detailed written description of the cooking procedure and consumed directly after preparation (see above Evening test and reference meals). After the evening meals, the subjects were fasting until the breakfast was served the subsequent morning at the research department. The subjects arrived to the department at 0745 am. An intravenous cannula (BD Venflon, Becton Dickinson) was inserted into an antecubital vein for blood sampling. Fasting (f-) blood samples were collected and appetite and breath H_2_ registered before the breakfast. Test variables were determined repeatedly in the postprandial period after breakfast and lunch according to time intervals previously stated. The breakfast was consumed at 0800 am and finished within 15 min. Coffee/tea (without milk or sugar) or water (200 ml) were served at 120 min after the breakfast, each subject keeping the same drink throughout the study. The lunch was served 210 min after commencing the breakfast. The amounts of breakfast and lunch consumed were registered. During the experimental days (5.5 h) the subjects were told to maintain a constant, low physical activity, preferably reading or similar.

### Analysis of nutrient composition in evening meals, breakfast and lunch

The test- and reference meals were analysed with respect to total starch [30], available starch [31], RS [32], and DF [33]. Information regarding content of starch and DF in evening test- and reference meals is provided in Table 1. Prior to analysis of total- and available starch, and DF the products were air dried and milled. RS was analysed on products as eaten. Available starch in the BK meal was calculated by subtracting RS from total starch, whereas potentially available starch content in the reference WWB and the commercial white wheat breakfast bread was analysed according to Holm *et. al*[31].

**Table 1:** **Composition of the evening test and reference meals**^**1**^ (% dry matter and g/serving, respectively)

**Meals**	**Portion weight**	**Total starch**	**RS**	**Available starch**	**Insoluble DF**	**Soluble DF**	**Total DF**	**RS+DF**
	*% dry matter*							
BK^2^	-	68.79	11.6	57.23	8.11	5.80	13.9	25.5
WWB	-	81.09	1.85	79.24	4.03	1.16	5.19	7.04
	*g/serving*							
BK^2^	96.8^3^	60.10	10.1	50	7.09	5.06	12.2	22.3
WWB	119.7	51.34	1.17	50^4^	2.29	0.66	2.95	4.12

^1^ Data are presented as means ± SEM. Available starch calculated by difference between total starch and RS. Values of total and available starch are based on means of 2 replications, RS means of 6 replications, DF means of 3 replications. BK, barley kernel; DF, dietary fibre; RS, resistant starch; WWB, white wheat bread.

^2^ Analyzed as eaten.

^3^ Portion size is based on raw barley kernels.

^4^ Analyzed according to Holm *et. al* (1986) [31].

The nutritional composition of the breakfast and lunch meals is displayed in Table 2. Prior to analysis, the breakfast sandwiches were prepared as eaten and then cut into small pieces and freeze dried. The lunch (hash) was prepared according to instructions and then mixed with addition of water into a paste, followed by freeze drying. Freeze dried samples were ground in a mortar prior to analysis. Samples were analysed for carbohydrates (available starch) [31], protein and fat. Crude protein content was determined using an elemental analyzer (FlashEA 1112, Thermo Fisher Scientific Inc, Waltham, MA, USA). Fat content was measured gravimetrically using the Schmid-Bondzynski-Ratzlaff (SBR) method.

**Table 2:** **Nutritional composition of the breakfast and lunch**^**1**^

	**Carbohydrates**^**2**^	**Protein**	**Fat**	**Energy content**
	*% dry matter*			*kcal*
Breakfast	62.6	11.9	13.6	59.8 /sandwich
Lunch	38.5	13.9	27.5	234/100 g
Ketchup^3^	-	-	-	85/100 g

^1^ The breakfast consisted of commercial white wheat bread with butter and ham and the lunch consisted of Swedish hash, composed of a mix of fried diced potato, meat and onion with the voluntary addition of ketchup. Values of carbohydrate and fat are based on means of 2 replications, protein means of 4 replications and fat means of 2 replications.

^2^ Analyzed as available starch according to Holm *et. al* (1986) [31].

^3^ Values obtained from the manufacturer.

### Analysis of physiological variables

Finger-prick capillary blood samples were taken for determination of blood glucose (HemoCue®B-glucose, HemoCue AB, Ängelholm, Sweden). Venous blood was collected for measurement of serum (s) FFA and s-IL-6, and plasma (p) adiponectin, p-insulin, p-ghrelin, p-GIP and p-GLP-1.

Milliplex™ MAP (HMH-34K Milliplex™ MAP, Millipore, St.Charles, USA) analyses were performed for simultaneous measurement of insulin, active ghrelin, total GIP, and active GLP-1. Blood collecting tubes for analysis with Milliplex™ MAP were added with an inhibition cocktail consisting of DPPIV-inhibitor (10 μl/ml blood) (Millipore, St Charles, USA) and Pefablock SC (1 mg/ml blood) (Roche Diagnostics, Mannheim, Germany) prior to blood sampling. Tubes containing inhibition cocktail were kept cold for maximum 6 days until blood sampling. After blood collection the tubes intended for Milliplex™ MAP analyses were centrifuged within 30 minutes at 1000 × g for 10 minutes in 4°C. The plasma was removed and immediately stored (-20°C) in Eppendorf-tubes until analysis. The blood samples were analysed using immunoassays on the surface of fluorescently labelled microsphere beads and read on the Luminex 200 instrument (Luminex Corporation, USA). Milliplex™ Analyst v.3.4 (VigeneTech Inc., Carlisle, USA) was used for the evaluation of the results.

Plasma and serum for analysis of FFA, IL-6 and adiponectin were allowed to clot in ambient temperature (serum) or kept on ice and centrifuged within 30 minutes (plasma). Samples were separated (3500 rpm for 10 min in 4°C) and stored in a freezer (-20°C) until analysed. FFA concentrations were determined with an enzymatic colorimetric method (NEFA C, ACS-ACOD method, WAKO Chemicals GMbH, Germany). IL-6 was determined with a high sensitivity solid-phase immunoassay kit (R&D Systems Inc, Minneapolis, USA). Adiponectin concentrations were measured with a solid phase 2-site enzyme immunoassay kit (Mercodia Adiponectin ELISA, Mercodia, Uppsala, Sweden). Hydrogen in expired air was measured as an indicator of colonic fermentation using an EC 60 or Gastro+ (Bedfont EC60 Gastrolyzer, Rochester, England).

### Calculations and statistical methods

Data are expressed as means ± SEM. The incremental area- and area under the curve (iAUC and AUC, respectively) was calculated for each subject and test meal, using the trapezoid model. iAUC or AUC were used in the statistical evaluation of results regarding blood glucose, insulin, GLP-1, GIP, ghrelin and appetite. Incremental peak (iPeak) concentrations were calculated for glucose and insulin as individual maximum postprandial increase from baseline. For calculation of incremental responses, the specific fasting value at the day for postprandial measurements was used. GraphPad Prism (version 4 and 5, GraphPad Software, San Diego, CA, USA) was used for graph plotting and calculation of AUC. Significant differences in test variables after the different test meals were assessed with ANOVA (general linear model), in MINITAB Statistical Software (release 14; Minitab, Minitab Inc, State College, PA). In the cases of unevenly distributed residuals (tested with Anderson-Darling and considered unevenly distributed when *P* < 0.05), Box Cox transformation were performed on the data prior to the ANOVA. Differences between the products at different time points were evaluated using a mixed model (PROC MIXED in SAS release 9.2; SAS Institute Inc, Cary, NC) with repeated measures and an autoregressive covariance structure. Randomization of the test products were performed in MINITAB Statistical Software (release 14; Minitab, Minitab Inc, State College, PA). If the value from a test subject were missing for one of the products, the test subject was excluded from that specific calculation.

For breath hydrogen where the variation in the concentration scarcely changed over time, a weighted mean were produced by calculating a mean for equal time intervals (1 mean per hour) over the test period, and then a mean for the different hours were calculated and used in statistical analysis. One subject failed to follow instructions at voluntary food intake and data from this variable and subject is therefore excluded from the statistical analysis (n = 18). Due to analytical difficulties two subject are withdrawn from blood glucose calculations (n = 17). The glucose profile (GP^2^) is calculated as the time (min) during which the blood glucose are above fasting concentration divided with the squared incremental peak value (mM) of blood glucose for each test subject and test meal. In the cases where the blood glucose concentration remained above fasting for the entire 210 min, the duration value was set to 210 min. GP^2^ is used as a measure of the course of glycaemia including also characteristics in the late postprandial phase, high values are considered beneficial [34]. *P*-values < 0.05 were considered statistically significant.

## Results

### Breath H_2_

**Figure 1:**
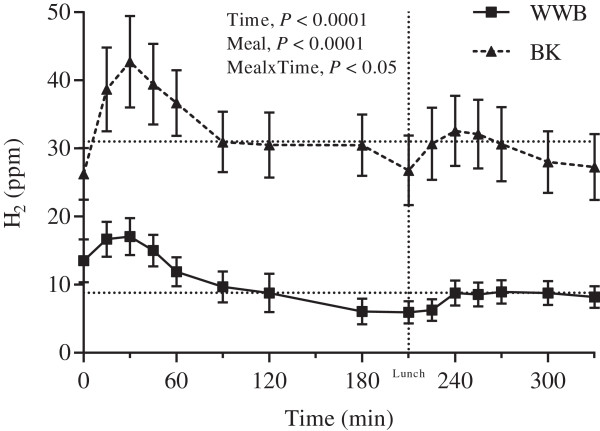
**Breath H**_**2 **_**excretion during the experimental day.** Mean postprandial H_2_ excretion 10.5-16 h post ingestion of evening meals with BK or WWB respectively. Values are means ± SEM. Dotted lines at the y-axes indicate weighted mean calculated for WWB (8.8 ppm) and BK (31 ppm), respectively. BK, barley kernel; H_2_, breath hydrogen; WWB, white wheat bread.

The f-breath H_2_ excretion (*P* < 0.01) and the mean breath H_2_ excretion during the experimental day (0–330 min, *P* < 0.001) was significantly elevated after BK as a late evening meal compared to the WWB reference meal (Table 3–4, Figure 1).

**Table 3:** **Physiological responses, breath H**_**2 **_**and subjective appetite scores at fasting, 10.5 h after evening test or reference meal**^**1**^

**Variables**		**WWB**	**BK**	**%**^**2**^
Glucose	*mmol/L*	5.2 ± 0.1	5.3 ± 0.1^3^	2
Insulin	*pg/mL*	607 ± 78	600 ± 58	-1
GLP-1	*pg/mL*	57.1 ± 8.2	68.7 ± 9.1 *	20
GIP	*pg/mL*	26.5 ± 2.1	29.5 ± 2.4	11
Ghrelin	*pg/mL*	108 ± 12.7	91.1 ± 9.3^3^	-16
FFA	*mmol/L*	0.38 ± 0.03	0.31 ± 0.03 *	-18
IL-6	*pg/mL*	0.60 ± 0.07	0.49 ± 0.03^4^	-18
Adiponectin	*μg/mL*	9.3 ± 0.5	9.2 ± 0.5	-1
H_2_	*ppm*	16.5 ± 2.0	24.7 ± 2.2 **	50
Satiety	*mm*	18.8 ± 2.7	22.8 ± 3.1	21
Hunger	*mm*	60.3 ± 3.4	61.8 ± 4.3	2

^1^ Values are means ± SEM. *Different from WWB *P* < 0.05; **P < 0.01. BK, barley kernel; GIP, glucose-dependent insulinotropic polypeptide; GLP-1, glucagon-like peptide-1; H_2_, breath hydrogen; WWB, white wheat bread.

^2^ The percentage change is calculated as the difference from the WWB to the BK.

^3^*P* = 0.07.

^4^*P* = 0.06.

**Table 4:** **Appetite sensations, breath H**_**2 **_**and energy intake**^**1**^ after breakfast and lunch, following evening test- or reference meal^1^

**Test variables**			**WWB**	**BK**	**%**^**2**^
Hunger	AUC 0–120 min	*min⋅mm*	3 330 ± 343	2 960 ± 372	-11
Hunger	AUC 120–210 min	*min⋅mm*	4 750 ± 380	4 090 ± 395*	-14
Hunger	AUC 0–330 min	*min⋅mm*	10 800 ± 892	9 510 ± 997*	-12
Satiety	AUC 0–120 min	*min⋅mm*	6 130 ± 384	6 510 ± 365	6
Satiety	AUC 120–210 min	*min⋅mm*	2 780 ± 425	3 180 ± 342	14
Satiety	AUC 0–330 min	*min⋅mm*	15 810 ± 1060	16 540 ± 917	5
Desire to eat	AUC 0–120 min	*min⋅mm*	3 600 ± 413	3 310 ± 488	-8
Desire to eat	AUC 120–210 min	*min⋅mm*	5 000 ± 433	4 530 ± 477	-10
Desire to eat	AUC 0–330 min	*min⋅mm*	11 700 ± 1 140	10 800 ± 1 220	-8
H_2_	Mean 0–120 min	*ppm*	11 ± 2.1	33 ± 4.4***	200
H_2_	Mean 210–330 min	*ppm*	7.9 ± 1.3	29 ± 4.7***	267
H_2_	Mean 0–330 min	*ppm*	8.8 ± 1.5	31 ± 4.4***	248
Energy intake	Breakfast	*kcal*	415 ± 38.0	400 ± 30.7	-4
Energy intake	Lunch	*kcal*	804 ± 98.4	709 ± 74.0*	-12
Energy intake	Cumulative	*kcal*	1 220 ± 130	1 110 ± 93.2^3^	-9

^1^ Values are means ± SEM. *Different from WWB *P* < 0.05; ***P < 0.001. BK, barley kernel; AUC, area under curve; H_2_, breath hydrogen; WWB, white wheat bread.

^2^ The percentage change is calculated as the difference from the WWB to the BK.

^3^*P* = 0.07.

### Voluntary energy intake at breakfast and lunch

Ad libitum energy intake at breakfast was 400 kcal and 415 kcal following BK and WWB evening meal, respectively, with no significant difference depending on the previous evening meal (Table 4). When given BK as a late evening meal the test subjects significantly reduced their energy intake at lunch by 12%, compared to energy intake at lunch after the WWB evening meal (*P <* 0.05) (Table 4). The cumulative energy intake over breakfast and lunch tended to be lower after boiled BK as compared to after the WWB evening meal (-9%) (*P* = 0.07)

### Glucose and insulin

**Figure 2:**
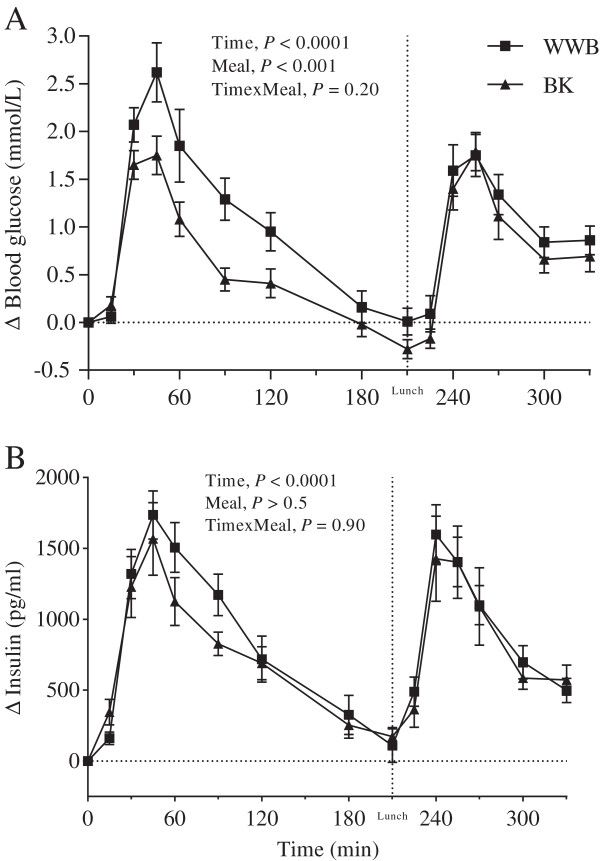
**Incremental blood glucose and plasma insulin response.** Mean incremental blood glucose (**A**) and plasma insulin (**B**) changes (Δ) from fasting concentrations after evening meals with BK or WWB, respectively. Values are means ± SEM. BK, barley kernel; WWB, white wheat bread.

No differences were seen in f-glucose- or f-insulin concentrations depending on previous evening meals (Table 3). The BK evening meal reduced the glucose iAUC during the course of the entire experimental day (0–330 min, *P* < 0.01), compared with the WWB evening meal. After breakfast there was also a reduced glucose iPeak following BK as an evening meal (*P* < 0.001), as compared to WWB (Figure 2). In addition, the GP^2^ value was higher after breakfast post the evening meal with BK (*P* < 0.05) (Table 5). No significant differences were observed in the post-prandial response of insulin depending on evening meals (Figure 2, Table 5).

**Table 5:** **Results for blood glucose and serum insulin**^**1**^ after breakfast and lunch, following evening test- or reference meal^1^

**Test variables**			**WWB**	**BK**	**%**^**2**^
Glucose	iPeak breakfast	*mmol/L*	3.0 ± 0.3	2.0 ± 0.2***	-33
Glucose	iAUC 0–120 min	*mmol⋅min/L*	168 ± 22	99 ± 10***	-41
Glucose	iPeak lunch	*mmol/L*	2.2 ± 0.2	2.0 ± 0.2	-7
Glucose	iAUC 210–330 min	*mmol⋅min/L*	126 ± 15	107 ± 13	-15
Glucose	iAUC 0–330 min	*mmol⋅min/L*	341 ± 36	227 ± 24**	-34
Glucose	GP^2^	*min/mmol*^*2*^	24.6 ± 4.1	39.0 ± 4.8*	59
Insulin	iPeak breakfast	*pg/L*	2.2 ± 0.2	1.7 ± 0.2	-16
Insulin	iAUC 0–120 min	*pg⋅min/L*	125 ± 11	107 ± 13	-15
Insulin	iPeak lunch	*pg/L*	1.8 ± 0.2	1.7 ± 0.3	-3
Insulin	iAUC 210–330 min	*pg⋅min/L*	103 ± 9	100 ± 18	-3
Insulin	iAUC 0–330 min	*pg⋅min/L*	276 ± 26	241 ± 33	-13

^1^ Values are means ± SEM. *Different from WWB *P* < 0.05; **P < 0.01; ***P < 0.001. BK, barley kernel; GP^2^, glucose profile; iAUC, incremental area under curve; iPeak, incremental peak; WWB, white wheat bread.

^2^ The percentage change is calculated as the difference from the WWB to the BK.

### FFA

The BK evening meal generated lower concentrations of circulating f-FFA compared to the WWB (*P <* 0.05) (Table 3). No significant differences were observed prior to lunch (210 min) in FFA depending on the preceding evening meal (BK 0.20 ± 0.03; WWB 0.21 ± 0.04 mmol/L).

### Incretin hormones and ghrelin

**Figure 3:**
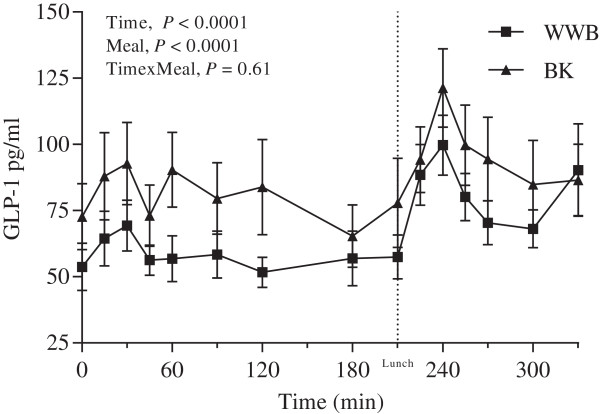
**GLP-1 response.** Mean concentration of plasma GLP-1 during the experimental day following evening meals with BK or WWB, respectively. Values are means ± SEM. BK, barley kernel; GLP-1, glucagone-like peptide-1; WWB, white wheat bread.

**Figure 4:**
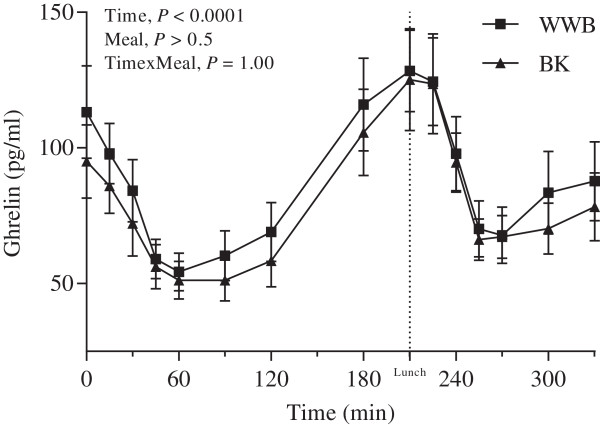
**Ghrelin response after breakfast.** Mean concentration of plasma ghrelin post ingestion of evening meals with BK or WWB, respectively. Values are means ± SEM. BK, barley kernel; WWB, white wheat bread.

The GLP-1 concentrations were significantly increased at fasting (*P <* 0.05, Table 3) and during the whole experimental period (AUC 0–330 min, *P* < 0.01, Figure 3, Table 6), after BK as compared to WWB evening meal. Significantly raised concentrations of GIP were observed in the postprandial period 60–120 min (AUC) after breakfast post BK evening meal (12.8 ± 1.0 pg⋅min/L) as compared to after WWB evening meal (10.7 ± 0.9 pg⋅min/L) (*P* < 0.05). At fasting a trend (*P* = 0.07) was observed toward decreased concentration of plasma ghrelin after BK as compared to WWB evening meal. No significant differences were detected in the post-prandial ghrelin response (Table 6, Figure 4).

**Table 6:** **Plasma GLP-1, GIP and ghrelin response**^**1**^ after breakfast and lunch, following evening test- or reference meal^1^

**Test variables**			**WWB**	**BK**	**%**^**2**^
GLP-1	AUC 0–120 min	*pg⋅min/L*	7.7 ± 0.9	11.0 ± 1.7**	43
GLP-1	AUC 210–330 min	*pg⋅min/L*	10.0 ± 1.2	12.5 ± 1.8*	25
GLP-1	AUC 0–330 min	*pg⋅min/L*	23.0 ± 2.6	30.7 ± 4.3**	34
GIP	AUC 0–120 min	*pg⋅min/L*	18.2 ± 1.5	21.2 ± 1.5	16
GIP	AUC 210–330 min	*pg⋅min/L*	33.3 ± 3.5	29.1 ± 2.2	-12
GIP	AUC 0–330 min	*pg⋅min/L*	62.9 ± 5.4	62.6 ± 4.6	-0.5
Ghrelin	AUC 0–120 min	*pg⋅min/L*	8.4 ± 1.1	7.4 ± 0.9	-12
Ghrelin	AUC 210–330 min	*pg⋅min/L*	10.4 ± 1.5	10.0 ± 1.1	-4
Ghrelin	AUC 0–330 min	*pg⋅min/L*	28.1 ± 3.7	25.9 ± 3.0	-8

^1^ Values are means ± SEM. *Different from WWB *P* < 0.05; **P < 0.01. AUC, area under curve; BK, barley kernel; GIP, glucose-dependent insulinotropic polypeptide; GLP-1, glucagon-like peptide-1; WWB, white wheat bread.

^2^ The percentage change is calculated as the difference from the WWB to the BK.

### Inflammatory variables

**Figure 5:**
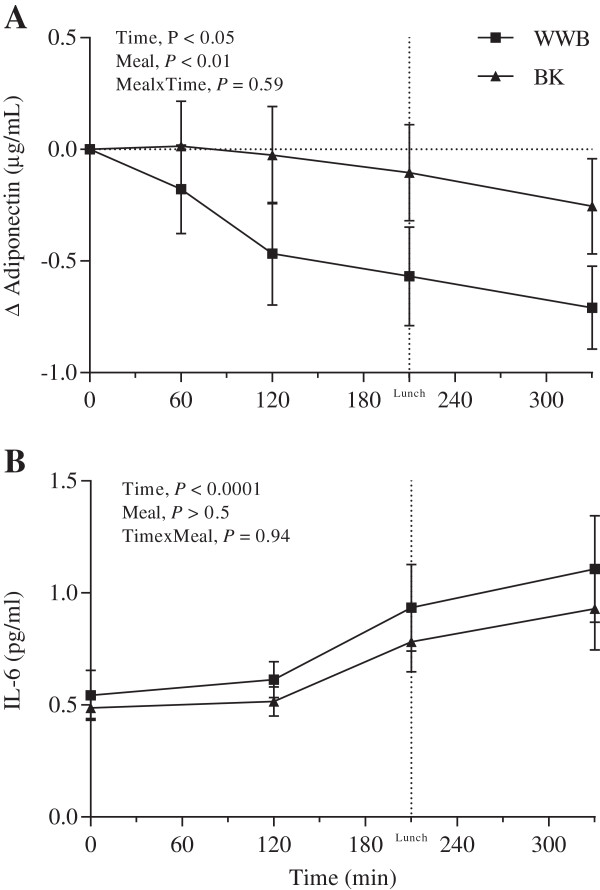
**Incremental adiponectin changes and IL-6 response.** Mean incremental plasma adiponectin (**A**) changes (Δ) and mean concentration of serum IL-6 (**B**) during the experimental day following evening meals with BK or WWB, respectively. Values are means ± SEM. BK, barley kernel; IL-6, interleukin-6; WWB, white wheat bread.

There was a tendency towards decreased f-IL-6 concentrations after BK evening meal (*P* = 0.06) (Table 3). The postprandial concentration of IL-6 is shown in Figure 5. There were no significant differences in the fasting state of adiponectin depending on the previous evening meal. However, a less pronounced decrease in plasma adiponectin concentrations from fasting state (0–330 min) was observed after BK as an evening meal (-1.4%) compared to after WWB evening meal (-7.9%) (*P* < 0.05) (Figure 5).

### Subjective appetite ratings

**Figure 6:**
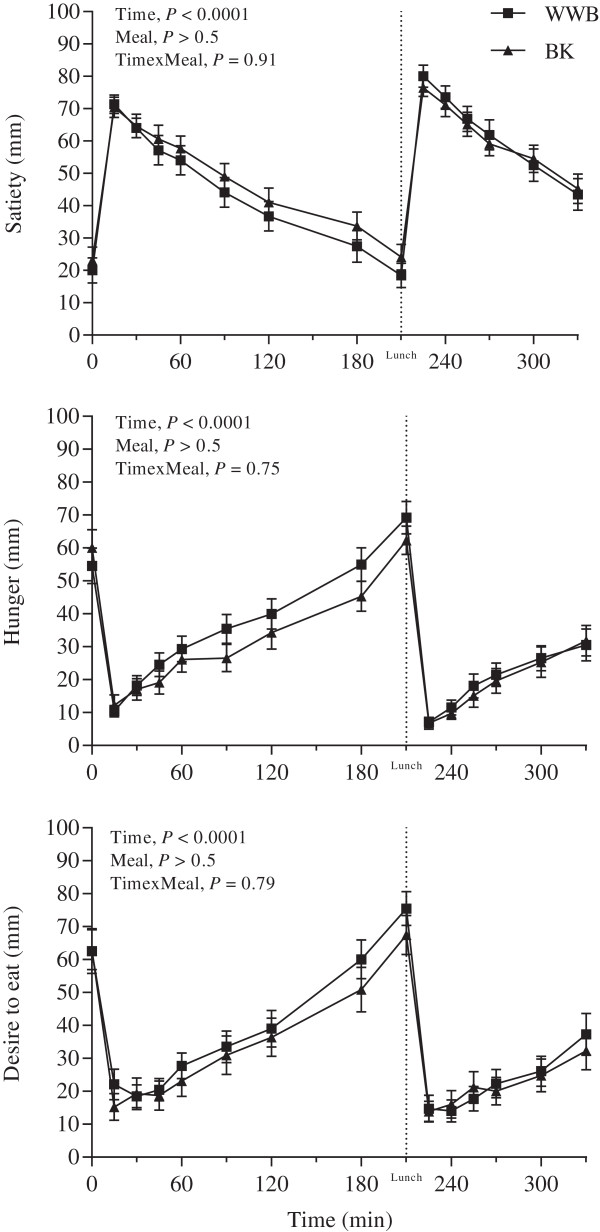
**Post-prandial responses of satiety, hunger and desire to eat.** Mean subjective ratings of appetite (VAS) during 5.5 h after breakfast and lunch, following an evening meal of BK or WWB, respectively. Values are means ± SEM. BK, barley kernel; WWB, white wheat bread.

Postprandial results for subjective appetite ratings are presented in Figure 6 and Table 4. No significant differences were measured in subjective appetite ratings in the fasting state depending on the previous evening meal (Table 3). There was a lower *feeling of hunger* at breakfast and lunch (AUC 0–330 min) after BK evening meal, as compared to the WWB evening meal (*P <* 0.05). Also, the BK evening meal generated attenuated *feeling of hunger* in the *late* postprandial period after breakfast as compared to the WWB (AUC 120–210 min) (*P <* 0.05).

## Discussion

The purpose of the present study was to examine the metabolic effects of intrinsic fermentable carbohydrates in boiled barley kernels in an over-night perspective. In comparison to previous studies [23,35], the experimental design is novel in that the experimental sampling period is significantly extended, covering a period ranging from 10.5-16 h after ingestion of test and reference evening meal. Further the present study aimed to investigate the influence of evening meals on metabolic test variables, appetite, and voluntary energy intake following ad libitum breakfast- and lunch meals; that is in a more realistic eating situation than studies with standardized meal size.

Previous studies have revealed that dietary supplementation of inulin and oligofructan during two weeks [28], or consumption of other specific indigestible carbohydrates, e.g. present in BK, as a single late evening meal [23,36], decrease the glucose response and increase gut microbial activity (as determined by breath H_2_ and/or plasma short chain fatty acids (SCFA)) at the following breakfast meal in healthy humans. In accordance with previous findings, the present study revealed decreased glucose response after breakfast following BK as an evening meal compared to WWB, and increased gut microbial fermentation, as indicated by elevated breath H_2_ concentrations. As a novel finding, the BK evening meal decreased glucose iAUC during the course of the entire experimental day (0–330 min), and increased GP^2^ after breakfast, all indicative of smoother postprandial glucose excursion. In this context it must be noted that in contrast to the standardized meal size previously used, glucose measurements in the present study were conducted after ad libitum food intake (standardized in quality). At breakfast there was only a minor (4%, non-significant) decrease in energy intake after BK. It is therefore highly unlikely that the differences seen in metabolic variables after breakfast could be explained by differences in energy intake at breakfast. However, the glucose responses at lunch following the BK evening meal may, at least to a minor degree, have been affected by the reduced caloric intake at lunch.

It has been hypothesized that modulation of gut microbiota by oligofructans, may interfere beneficially with host metabolism. Thus, dietary supplementation with oligofructose for 14 weeks decreased glucose AUC and voluntary energy intake, as well as reduced inflammatory tonus in mice models [21]. Studies in rodents have further shown that oligofructose feeding during 4–5 weeks may promote epithelial L-cell differentiation in the gut, contributing to higher GLP-1 production [20,37]. It has been demonstrated that SCFA produced by bacterial fermentation may trigger signaling cascades through acting on SCFA receptors on L-cells (in vitro model), resulting in increased release of gut peptides such as GLP-1 and Peptide YY (PYY) [38]. Fructan supplementation during two weeks was also reported to reduce glucose response, reduce *feeling of hunger* and increase GLP-1 concentration in response to an ad libitum meal in humans [28]. Less is known about the gut mediated effects of intrinsic indigestible carbohydrates in food, e.g. boiled barley kernels, on glycaemia, gut fermentation, and appetite regulation, and to the authors’ knowledge, data from human studies are scarce. Nilsson *et al.* (2008) found inverse correlations between glucose iAUC and breath H_2,_ supporting a link between colonic fermentation and glucose regulation [23]. A particularly interesting observation in the present study is a 34% increase in plasma concentration of GLP-1 in the morning (0–330 min) after the BK evening meal, compared to the evening meal with WWB. In addition, in the time period 60–120 min after breakfast also GIP concentrations (AUC) were increased after BK, which is in accordance with previous findings in our research group [23]. Both GLP-1 and GIP have implications in glucose homeostasis, and in addition GLP-1 is considered as a modulator of appetite regulation. Both glucose intolerance and obesity seems to be related to decreased levels of GLP-1, independently of one another [27]. In the present study, the BK evening meal decreased the *feeling of hunger* during the whole experimental day, as compared to the evening WWB, and reduced voluntary energy intake (-12%) at lunch. Previously it has been found that intravenous infusions of GLP-1 (50 pmol/kg⋅h) during 4 h reduced voluntary energy intake by 12% at a subsequent lunch meal, as compared to saline in young healthy men [39]. The results in the present study are thus in accordance with previous studies showing that GLP-1 reduce food intake and increase satiety in both lean and obese subjects [40]. Although not significant, there was a trend (*P* = 0.07) to reduced fasting levels of ghrelin after the BK evening meal by 16% and there was a, non-significant, 10% reduction of ghrelin in the late post-prandial phase prior to lunch (AUC 120–210 min) as compared to after WWB. Intravenous administration during 4 h of the orexigenic hormone ghrelin, demonstrates increased food intake in healthy subjects at a subsequent meal [41]. A connection has been proposed between colonic fermentation and reduced concentrations of serum ghrelin in healthy subjects, 6 h after ingestion of inulin, possible mediated through the formation of SCFA [42]. It can be suggested that the effects on gastro-intestinal hormones seen in the present study after the BK evening meal, stem from colonic fermentation of indigestible DF in the BK. GLP-1 and GIP, are known to exert insulinotropic effects [43] and have been suggested to account for up to 70% of meal induced insulin release in humans [44]. Noticeably, the lowered glycaemia and increased incretin concentrations observed during the experimental day after BK evening meal were not accompanied by increased insulin response. However, GLP-1 has previously been reported to improve insulin sensitivity in T2D [45], a phenomenon that might have contributed to the facilitated glucose regulation during the experimental day in the present study in healthy subjects. Another factor that might have contributed to an improved insulin sensitivity after the BK evening meal is reduced f-FFA concentrations compared to the evening WWB reference meal [46]. Belfort *et al.* (2005) showed that even a modest increase in plasma FFA, well within the physiological range, causes a dose-dependent inhibition of insulin-stimulated glucose disposal and insulin signaling in healthy lean subjects [47].

Inulin and oligofructan supplementation during two weeks, was also reported to reduce glucose response, reduce *feeling of hunger* and increase GLP-1 concentration in response to an ad libitum meal in humans [28]. Less is known about the gut mediated effects of intrinsic indigestible carbohydrates in food, e.g. boiled barley kernels, on glycaemia, gut fermentation, and appetite regulation, and to the authors’ knowledge, data from human studies are scarce. Nilsson *et al.* (2008) found inverse correlations between glucose iAUC and breath H_2_ supporting a link between colonic fermentation and glucose regulation [23]. A particularly interesting observation in the present study is a 34% increase in plasma concentration of GLP-1 in the morning (0–330 min) after the BK evening meal, compared to the evening meal with WWB. In addition, in the time period 60–120 min after breakfast also GIP concentrations (AUC) were increased after BK, which is in accordance with previous findings in our research group [23]. Both GLP-1 and GIP have implications in glucose homeostasis, and in addition GLP-1 is considered as a modulator of appetite regulation. Both glucose intolerance and obesity seems to be related to decreased levels of GLP-1, independently of one another [27]. In the present study, the BK evening meal decreased the *feeling of hunger* during the whole experimental day, as compared to the evening WWB, and reduced voluntary energy intake (-12%) at lunch. Previously it has been found that intravenous infusions of GLP-1 (50 pmol/kg⋅h) during 4 h reduced voluntary energy intake by 12% at a subsequent lunch meal, as compared to saline in young healthy men [39]. The results in present study are thus in accordance with previous studies showing that GLP-1 reduce food intake and increase satiety in both lean and obese subjects [40]. Although not significant, there was a trend (*P* = 0.07) to reduced fasting levels of ghrelin after the BK evening meal by 16% and there was a, non-significant, 10% reduction of ghrelin in the late post-prandial phase prior to lunch (AUC 120–210 min) as compared to after WWB. Intravenous administration during 4 h of the orexigenic hormone ghrelin, demonstrates increased food intake in healthy subjects at a subsequent meal [41]. A connection has been proposed between colonic fermentation and reduced concentrations of serum ghrelin in healthy subjects, 6 h after ingestion of inulin, possible mediated through the formation of SCFA [42]. It can be suggested that the effects on gastro-intestinal hormones seen in the present study after the BK evening meal, stem from colonic fermentation of indigestible DF in the BK.

GLP-1 and GIP, are known to exert insulinotropic effects [43] and have been suggested to account for up to 70% of meal induced insulin release in humans [44]. Noticeably, the lowered glycaemia and increased incretin concentrations observed during the experimental day after BK evening meal were not accompanied by increased insulin response. However, GLP-1 has previously been reported to improve insulin sensitivity in T2D [45], a phenomenon that might have contributed to the facilitated glucose regulation during the experimental day in the present study in healthy subjects. Another factor that might have contributed to an improved insulin sensitivity after the BK evening meal is reduced f-FFA concentrations compared to the evening WWB reference meal [46]. Belfort *et al.* (2005) showed that even a modest increase in plasma FFA, well within the physiological range, causes a dose-dependent inhibition of insulin-stimulated glucose disposal and insulin signaling in healthy lean subjects [47].

In the present study, f-IL-6 concentrations tended to be decreased (*P* = 0.06) after the BK evening meals, which is in accordance with previous studies [23,36]. Low-grade chronic systemic inflammation is associated with obesity and insulin resistance [48]. Anti-inflammatory properties of specific indigestible carbohydrates, as present in e.g. BK, might constitute a promising approach aiming at dietary prevention and/or treatment of obesity and the metabolic syndrome. In a cross-sectional study by Yannakoulia *et al.* (2008), consumption of WG cereals and low-fat dairy products were positively associated with adiponectin concentrations among healthy women [49]. The authors suggested that adiponectin may be a mediator of the decreased risk of T2D associated with higher WG intake. Adiponectin has been shown to possess not only insulin sensitizing properties but also to have anti-inflammatory effects [50]. Decreased concentrations of adiponectin are observed in obesity and T2D [51,52]. In the present study, we show that BK evening meal resulted in maintained adiponectin concentrations in the post-prandial period after breakfast as compared to the decrease observed after WWB evening meal. The metabolic relevance of this finding remains to be elucidated. However, adiponectin concentrations in subjects with metabolic disorders show a more pronounced decrease in the post-prandial phase compared to healthy subjects [53,54].

## Conclusions

The results indicate that indigestible carbohydrates, as present in BK, have the potential to facilitate glucose regulation in healthy subjects in a time period of 10.5-16 h, decrease inflammatory markers, decrease FFA, decrease hunger sensations and reduce energy intake at a subsequent lunch. Interestingly, the BK evening meal resulted in an increased release of GLP-1 during the whole experimental period. The effects are suggested to be mediated through gut microbial fermentation, proposing a role of gut microbiota in modulating host metabolism in humans. Colonic fermentation of specific indigestible carbohydrates may provide one possible mechanism by which WG have proven beneficial in prevention of obesity and T2D. Taken together, the beneficial effects of BK are supportive for a prebiotic potential of intrinsic indigestible carbohydrates in barley kernel based products.

## Abbreviations

AUC: Area under curve; BK: Barley kernel; BMI: Body mass index; DF: Dietary fibre; f-: Fasting-; GIP: Glucose-dependent insulinotropic polypeptide; GLP-1: Glucagon-like peptide-1; GP2: Glucose profile; H2: Breath hydrogen; iAUC: Incremental area under curve; IL-6: Interleukin-6; iPeak: Incremental peak; MetS: Metabolic syndrome; PYY: Peptide YY; RS: Resistant starch; SCFA: Short chain fatty acids; T2D: Type 2 diabetes; VAS: Visual analogue scale; WG: Whole grain; WWB: White wheat bread

## Competing interests

The authors declare that they have no competing interests.

## Authors´ contributions

ACN, EMÖ, and IMEB designed the research; EVJ, ACN, EMÖ, and IMEB supervised and/or conducted the research; EVJ analyzed data and performed statistical analysis; EVJ, ACN, EMÖ, and IMEB wrote the paper; and ACN, EMÖ and IMEB had primary responsibility for the final content. All authors have read and approved the final manuscript.
